# Veno-Venous Extracorporeal Membrane Oxygenation in Minipigs as a Robust Tool to Model Acute Kidney Injury: Technical Notes and Characteristics

**DOI:** 10.3389/fmed.2022.866667

**Published:** 2022-04-28

**Authors:** Antal Szabó-Biczók, Gabriella Varga, Zoltán Varga, Gábor Bari, Gyöngyvér Vigyikán, Ámos Gajda, Noémi Vida, Ádám Hodoniczki, Attila Rutai, László Juhász, Anna Nászai, Máté Gyöngyösi, Sándor Turkevi-Nagy, Dániel Érces, Mihály Boros

**Affiliations:** ^1^Division of Cardiac Surgery, Second Department of Internal Medicine and Cardiology Center, University of Szeged, Szeged, Hungary; ^2^Institute of Surgical Research, University of Szeged, Szeged, Hungary; ^3^Department of Pathology, University of Szeged, Szeged, Hungary

**Keywords:** extracorporeal membrane oxygenation, kidney injury, renal artery flow, mitochondrial function, ischemia, inflammation

## Abstract

**Objective:**

Veno-venous extracorporeal membrane oxygenation (vv-ECMO) can save lives in severe respiratory distress, but this innovative approach has serious side-effects and is accompanied by higher rates of iatrogenic morbidity. Our aims were, first, to establish a large animal model of vv-ECMO to study the pathomechanism of complications within a clinically relevant time frame and, second, to investigate renal reactions to increase the likelihood of identifying novel targets and to improve clinical outcomes of vv-ECMO-induced acute kidney injury (AKI).

**Methods:**

Anesthetized Vietnamese miniature pigs were used. After cannulation of the right jugular and femoral veins, vv-ECMO was started and maintained for 24 hrs. In Group 1 (*n* = 6) ECMO was followed by a further 6-hr post-ECMO period, while (*n* = 6) cannulation was performed without ECMO in the control group, with observation maintained for 30 h. Systemic hemodynamics, blood gas values and hour diuresis were monitored. Renal artery flow (RAF) was measured in the post-ECMO period with an ultrasonic flowmeter. At the end of the experiments, renal tissue samples were taken for histology to measure myeloperoxidase (MPO) and xanthine oxidoreductase (XOR) activity and to examine mitochondrial function with high-resolution respirometry (HRR, Oroboros, Austria). Plasma and urine samples were collected every 6 hrs to determine neutrophil gelatinase-associated lipocalin (NGAL) concentrations.

**Results:**

During the post-ECMO period, RAF dropped (96.3 ± 21 vs. 223.6 ± 32 ml/min) and, similarly, hour diuresis was significantly lower as compared to the control group (3.25 ± 0.4 ml/h/kg vs. 4.83 ± 0.6 ml/h/kg). Renal histology demonstrated significant structural damage characteristic of ischemic injury in the tubular system. In the vv-ECMO group NGAL levels, rose significantly in both urine (4.24 ± 0.25 vs. 2.57 ± 0.26 ng/ml) and plasma samples (4.67 ± 0.1 vs. 3.22 ± 0.2 ng/ml), while tissue XOR (5.88 ± 0.8 vs. 2.57 ± 0.2 pmol/min/mg protein) and MPO (11.93 ± 2.5 vs. 4.34 ± 0.6 mU/mg protein) activity was elevated. HRR showed renal mitochondrial dysfunction, including a significant drop in complex-I-dependent oxidative capacity (174.93 ± 12.7 vs. 249 ± 30.07 pmol/s/ml).

**Conclusion:**

Significantly decreased renal function with signs of structural damage and impaired mitochondrial function developed in the vv-ECMO group. The vv-ECMO-induced acute renal impairment in this 30-hr research protocol provides a good basis to study the pathomechanism, biomarker combinations or possible therapeutic possibilities for AKI.

## Introduction

Extracorporeal membrane oxygenation (ECMO) is a temporary, life-saving support therapy for patients with respiratory or circulatory failure. Two main configurations of ECMO are veno-arterial and veno-venous (vv-ECMO). Vv-ECMO provides respiratory support and alternative gas exchange when the possibilities for conventional mechanical ventilation are exhausted, and thus patients gain time in which the gas exchange function of the damaged lungs can return. If there is no improvement in lung function, further ECMO support is reasonable only if lung transplantation is indicated.

Despite the benefits of ECMO therapy, continuous extracorporeal circulation has several adverse effects, including bleeding, thrombosis and infections (1, 2). Many organ complications are also documented, but acute kidney injury (AKI) is perhaps the most frequent phenomenon, developing in 70–85% of ECMO patients (1, 3–6), with up to 78% of adults requiring vv-ECMO support (3, 7). It has also been shown that 45% of patients who develop renal failure require some modality of renal replacement therapy and that mortality is 3.7 times higher in these cases (6). Further, renal function may not return to normal in 58% of patients weaned from ECMO treatment following hospital discharge (4, 6), with 19% of patients possibly requiring some form of chronic renal replacement therapy (8).

A number of theories have been put forward to date, but the underlying pathophysiology of renal impairment is still not fully understood. The proposed mechanisms include hemodynamic changes, humoral and hormonal imbalance, and a combination of systemic inflammatory reactions and volume overload following the initiation of ECMO therapy (9). The main problem is that investigation of the effects of a vv-ECMO-induced process on undamaged kidneys is impossible in clinical scenarios, even though identification of elements involved in lung-related effects can help us to explain the controversial results and consequences. Therefore, the aim of our study was to develop a well-controlled large animal model that is suited to characterizing vv-ECMO-induced AKI and may contribute to the exploration of background pathophysiological processes. Such models can be useful to identify triggering factors or targeted specific therapies to prevent the development of EMCO-related AKI or to reduce severity, thus lowering its morbidity and mortality.

## Materials and Methods

The experiments were performed on female (*n* = 5) and castrated male (*n* = 7) outbred Vietnamese minipigs (*n* = 12; 46.5 ± 4.5 kg bw) in accordance with the National Institutes of Health guidelines on the handling of and care for experimental animals and EU Directive 2010/63 on the protection of animals used for scientific purposes (approval number: V.1480.2019).

### Animals and Anesthesia

Outbred Vietnamese minipigs (*n* = 12; weighing 46.5 ± 4.5 kg), obtained from a local, licensed breeder, were used. The animals were kept in the licensed, conventional hygienic level animal house of the Institute for an acclimatization period of 7–10 days with natural circadian light and free access to water and food. Prior to the experiments, the animals were fasted for 12 hrs with free access to tap water. At the beginning of the experiments, anesthesia was induced with an intramuscularly administered mixture of tiletamine-zolazepam (5 mg/kg im; Zoletil, Virbac, Carros, France) and xylazine (2 mg/kg im; Produlab Pharma, Raamsdonksveer, The Netherlands). After endotracheal intubation, mechanical ventilation was started with a tidal volume of 8–10 ml/kg, the respiratory rate was adjusted to maintain the end-tidal pressure of carbon dioxide in the 35–45 mmHg range, and the fraction of inspired oxygen (FiO_2_) was set to keep arterial partial pressure of oxygen (PaO_2_) between 80 and 100 mmHg. Anesthesia was maintained with a continuous infusion of propofol (6 mg/kg/h iv; Fresenius Kabi, Bad Homburg, Germany), midazolam (1.2 mg/kg/h; Torrex Chiesi Pharma, Vienna, Austria) and fentanyl (0.02 mg/kg/h; Richter Gedeon, Budapest, Hungary). Ringer’s lactate (RL) infusion was administered at a rate of 10 ml/kg/h. The depth of anesthesia was regularly controlled by monitoring the jaw tone and the absence of the interdigital reflex.

### Surgical Preparations

The anesthetized animals were placed in the supine position on a heating pad to maintain body temperature between 36 and 37°C. The left jugular vein was cannulated for fluid and drug administration, and the left femoral artery was cannulated for invasive hemodynamic monitoring and to measure cardiac output (CO) by transpulmonary thermodilution (PULSION Medical Systems, Munich, Germany). A urinary catheter was surgically placed in the bladder *via* the femoral incision.

To establish the ECMO circuit, the access cannula was inserted into the right femoral vein and the return cannula was introduced into the right jugular vein (21 Fr, HLS cannulas; Maquet, Rastatt, Germany). The position of the cannulas (access: proximal of the hepatic artery; return: orifice of the superior vena cava) was checked using an X-ray image intensifier (Ziehm SOLO; Ziehm Imaging GmbH, Nuremberg, Germany).

Before setting up the ECMO circuit, CO was directly measured with the PiCCO system *via* thermodilution, and the flow settings were initially adjusted accordingly at the start of ECMO circulation. Lung-safe ventilation was initiated during ECMO. Sweep gas and circuit blood flow rates were refined according to the arterial partial pressure of the carbon dioxide (PaCO_2_; 35–45 mmHg) and PaO_2_ (70–100 mmHg) values of the arterial blood gas samples, drawn from the left femoral artery. Transmembrane pressure was monitored, and the blood flow in the circuit was measured with the inline ultrasound flow probe of the pump. Mean arterial pressure (MAP) was monitored continuously and kept over 60 mmHg. As a positive inotropic treatment, norepinephrine was administered if necessary (0.05–0.35 μg/kg/h iv; Arterenol; Sanofi-Aventis, Frankfurt am Main, Germany).

The ECMO circulation was stopped after 24 h, and the ECMO cannulas were removed. Ventilation was set to the pre-ECMO settings. If necessary, oxygen and breathing rates were set according to the blood gas values.

After median laparotomy, the right renal artery was dissected free, and a perivascular flow probe was placed around it (Transonic Systems Inc., Ithaca, NY, United States) to measure the renal blood flow. The wound cut in the abdominal wall was then temporarily closed with clips.

### Experimental Protocol

Prior to the experiments, the animals underwent a general health check and if no outer injuries, discharge from body orifices or any signs of inflammations (swelling, edema, epithelial hyperemia) could be observed they were included in the study. The animals (*n* = 12) were randomly allocated into two experimental groups (*n* = 6, each group; the female-castrated male ratio: 0.5 for vv-ECMO and 0.33 for control group). In the vv-ECMO group, extracorporeal circulation was maintained for 24 h. After 24 h, ECMO was abandoned, the cannulas were removed, and the cannulation entry points were surgically closed. A further six-hour post-ECMO observation followed. For technical reasons, renal artery flow was measured in this phase only. In the control group, identical ECMO cannulation was completed, but extracorporeal circulation was not initiated. The same interventions and time frames were applied as in the vv-ECMO group. No animals were excluded from the study for any reasons.

During the 30-hour total observation, blood samples were taken every hour for blood gas analysis and for determination of total haemoglobin concentration (tHb) and hematocrit (Hct; Cobas b 123, Roche Ltd., Basel, Switzerland) and every 6 h (at baseline and hours 6, 12, 18, 24, and 30) to measure neutrophil gelatinase-associated lipocalin (NGAL) values. The urine was collected and measured hourly during the observation period to calculate the average hour diuresis, and further urine samples were taken for NGAL determination every 6 h (baseline and hours 6, 12, 18, 24, and 30).

**FIGURE 1:**
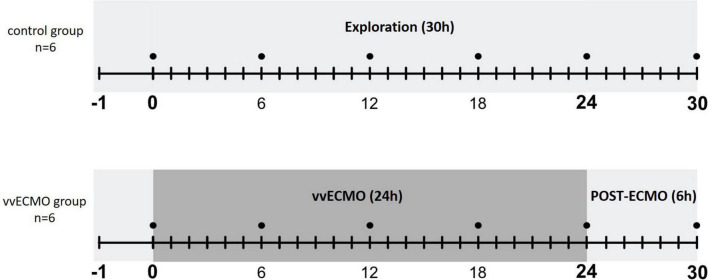
Scheme of experimental protocol.

At the end of the observation period, kidney tissue biopsies were taken to measure myeloperoxidase (MPO), xanthine oxidoreductase (XOR) enzyme activity and mitochondrial oxygen consumption as well as for histological examinations (Figure 1). After removal of tissue samples the animals were over anesthetized through the jugular vein cannula with a single 120 mg/kg dose of sodium pentobarbital (Sigma-Aldrich Inc, St. Louis, MO, United States).

### Hemodynamic Measurements

Mean arterial pressure and heart rate (HR) were monitored continuously and registered hourly, while transpulmonary thermodilution was used to measure CO hourly during the observation period (PiCCO Plus monitoring system; PULSION Medical Systems; Munich, Germany) and based on this CO value pulse contour analysis was used to continuously monitor (registered hourly) the stroke volume (SV). Blood flow signals (T206 Animal Research Flowmeter; Transonic Systems Inc., Ithaca, NY, United States) were recorded and registered every hour during the post-ECMO period with a computerized data acquisition system (SPELL Haemosys; Experimetria, Budapest, Hungary).

### Measurement of Mitochondrial Respiratory Function

Mitochondrial oxygen (O_2_) consumption (or volume-specific O_2_ flux) was assessed in kidney homogenates using High-Resolution FluoRespirometry (Oxygraph-2k, Oroboros Instruments, Innsbruck, Austria). After renal decapsulation, approx. 300 mg tissue samples were cut into smaller pieces, washed in phosphate-buffered saline (PBS) three times and then homogenized with a Potter–Elvehjem tissue grinder in Mir05 medium (pH 7.1).

Calibration and measurements were performed with continuous stirring (750 rpm) at 37°C in a 2 mL Mir05 respiration medium. After stabilization of the baseline respiration, NADH- and FADH_2_-supported LEAK respiration and complex I- and II-linked capacities of oxidative phosphorylation (OXPHOS) were measured in the presence of substrates (LEAK_GM_; 10 mmol/l glutamate, 2 mmol/l malate; and LEAK_S_; 10 mmol/l succinate) and saturating concentration of ADP (2.5 mmol/l). Rotenone (0.5 μmol/l; complex I inhibitor) was administered prior to succinate to block reverse electron transport-derived ROS formation and oxaloacetate accumulation (a known endogenous inhibitor of complex II). Following stimulation of OXPHOS, the integrity of the outer mitochondrial membrane was tested with exogenous cytochrome c (10 μmol/l).

Complex V (or ATP synthase) was inhibited by oligomycin (2.5 μmol/l) to evaluate LEAK respiration in a non-phosphorylating state (LEAK_Omy_). The respiratory control ratio (RCR), an index of coupling between respiration and phosphorylation, was expressed as a ratio of OXPHOS to the LEAK_Omy_ state. The electron transport system-independent respiration (or residual O_2_ consumption) was assessed following complex III inhibition with antimycin A (2.5 μmol/l).

The DatLab software (Oroboros Instruments, Innsbruck, Austria) was used for online display, respirometry data acquisition and analysis. Oxygen flux normalized to 8 mg wet weight expressed in pmol/s/ml.

### Kidney Histology

Kidney samples were fixed in 4% neutral buffered formalin. Tissue slices were embedded in paraffin using a Thermo Shandon PathCentre tissue processor. 3-μm-thick sections were cut with a Leica RM2125 rotary microtome. After deparaffinization and re-hydration, the sections were routinely stained for haematoxylin-eosin (HE) and periodic acid Schiff (PAS). The extent of tubular cell edema, apical cytoplasm vacuolization, tubular cell vacuolization, tubular lumen irregularity, loss of brush border, sloughing of tubular cells, tubular dilation, tubular cell necrosis, flattened and simplified tubular epithelium, and denudement of tubular basal membrane was assessed by a renal pathologist in a blinded way. To quantify these changes, we set up a kidney injury score, based on the extent of the histological changes noted above (0 points = 0%; 1 point = 1–20%; 2 points = 21–40%; 3 points = 41–60%; 4 points = 61–80%; and 5 points = 81–100%). The values of the tubular cell necrosis and denuded basement membranes were weighted by a factor of two.

### Measurement of Neutrophil Gelatinase Associated Lipocalin

Four-milliliter blood samples were drawn from the jugular vein into chilled polypropylene tubes containing EDTA (1 mg/mL) and 4-ml urine samples were collected in Eppendorf tubes at baseline and hours 6, 12, 18, 24 and 30. The blood samples were centrifuged at 1,200*g* for 10 min at 4°C. The plasma samples were then collected and stored at −70°C until assay. The urine samples were kept at −70°C until assay.

The plasma and urine concentration of NGAL was measured with a commercially available ELISA kit (Quantikine ELISA for Human Lipocalin-2/NGAL Immunoassay, United States R&D Systems, Minneapolis, MN, United States).

### Measurement of Tissue Xanthine-Oxidoreductase and Myeloperoxidase Enzyme Activities

Tissue samples were harvested immediately after the animals were sacrificed. A circular 1–2.5-cm-thick sample was excised from the distal pole of the left kidney. After saline rinsing, the sample was stored in liquid nitrogen for further analysis. Tissue biopsies kept on ice were homogenized in a phosphate buffer (pH 7.4) containing 50 mM Tris–HCl (Reanal, Budapest, Hungary), 0.1 mM EDTA, 0.5 mM dithiothreitol, 1 mM phenylmethylsulfonyl fluoride, 10 μg/ml soybean trypsin inhibitor and 10 μg/ml leupeptin (Sigma-Aldrich GmbH, Germany). The homogenate was centrifuged at 4°C for 20 min at 2,4000 *g*, and the supernatant was loaded into centrifugal concentrator tubes (Amicon Centricon-100; 100 000 MW cut-off ultrafilter).

Xanthine oxidoreductase activity was determined in the ultrafiltered, concentrated supernatant with a fluorometric kinetic assay based on the conversion of pterine to isoxanthopterine in the presence (total XOR) or absence (xanthine oxidase activity) of the electron acceptor methylene blue (10).

Myeloperoxidase activity was measured on the pellet of the homogenate (11). Briefly, the pellet was resuspended in a K_3_PO_4_ buffer (0.05 M; pH 6.0) containing 0.5% hexa-1,6-bis-decyltriethylammonium bromide. After three repeated freeze-thaw procedures, the material was centrifuged at 4°C for 20 min at 24,000 *g*, and the supernatant was used for MPO determination. Next, 0.15 ml of 3,3′,5,5′-tetramethylbenzidine (dissolved in DMSO; 1.6 mM) and 0.75 ml of hydrogen peroxide (dissolved in K_3_PO_4_ buffer; 0.6 mM) were added to 0.1 ml of the sample. The reaction led to the hydrogen peroxide-dependent oxidation of tetramethylbenzidine, which was detected spectrophotometrically at 450 nm (UV-1601 spectrophotometer; Shimadzu, Kyoto, Japan). MPO activity was measured at 37°C; then the reaction was halted after 5 min with the addition of 0.2 ml of H_2_SO_4_ (2 M). The data were expressed in terms of protein content.

### Statistical Analysis

Data analysis was performed with a statistical software package (SigmaStat for Windows; Jandel Scientific, Erkrath, Germany). Normality of data distribution was analyzed with the Shapiro–Wilk test. Friedman repeated-measures analysis of variance on ranks was applied within groups. Time-dependent differences from the baseline for each group were assessed by Dunn’s method. Differences between groups were analyzed with the Mann–Whitney test. Median values and 75th and 25th percentiles are provided in the figures; *P*-values <0.05 were considered significant. The sample sizes were estimated with PS: Power and Sample Size Calculation 3.1 software (12), and for sample size estimation the differences in the tissue XOR activity were the primary outcome measures.

## Results

### Changes in Systemic Hemodynamics

**FIGURE 2:**
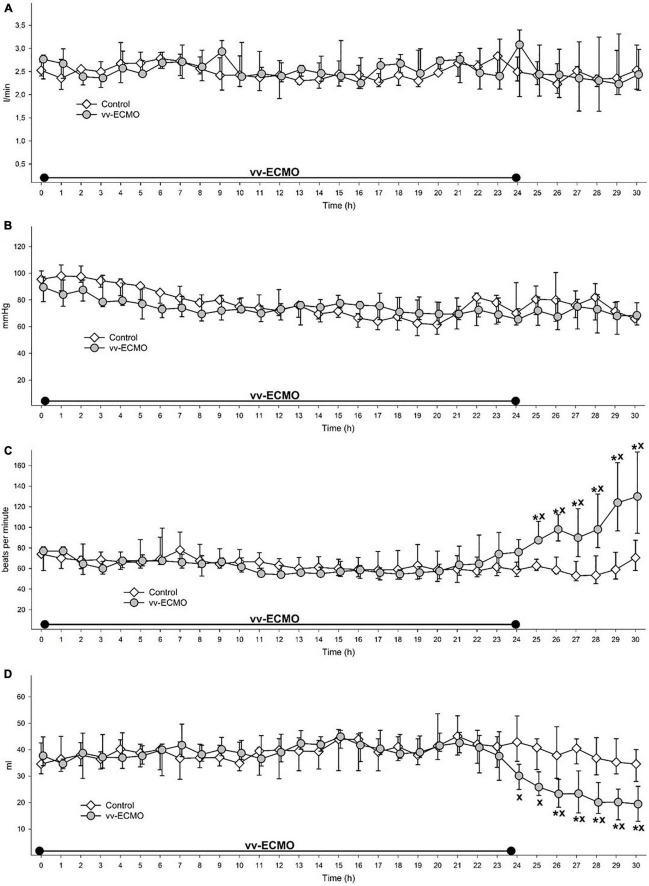
Changes in cardiac output **(A)**, mean arterial pressure **(B),** heart rate **(C)** and stroke volume **(D)** during the whole observation period in the control group (empty diamonds joined by a thin continuous line) and vv-ECMO group (gray circles joined by a continuous line). The plots demonstrate the median and the 25th (lower whisker) and 75th (upper whisker) percentiles. *P < 0.05 for the groups vs. baseline values (Friedman and Dunn tests). ^x^*P* < 0.05 for the vv-ECMO vs. control group values (Mann–Whitney test).

During the ECMO and post-ECMO period, we could not identify differences in CO (Figure 2A) and MAP between the two groups (Figure 2B). However, HR started to rise in the vv-ECMO group after 20 h and reached significantly higher values at 25 h compared to the control group (Figure 2C). The SV decreased significantly after 24 h compared to the control group (Figure 2D).

### Changes in Renal Hemodynamics and Function

**FIGURE 3:**
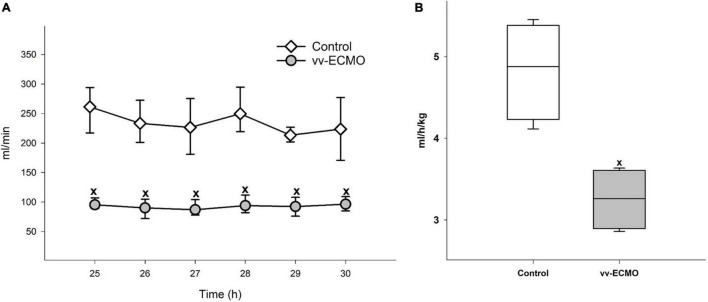
Changes in renal artery flow **(A)** in the control group (empty diamonds joined by a thin continuous line) and vv-ECMO group (gray circles joined by a continuous line). The plots demonstrate the median and the 25th (lower whisker) and 75th (upper whisker) percentiles. ^x^*P* < 0.05 for the vv-ECMO vs. control group values (Mann–Whitney test). Changes in average hour diuresis **(B)** in the control group (empty box) and vv-ECMO group (striped gray box). The plots demonstrate the median (horizontal line in the box), the 25th and 75th percentiles, and the range of data (whiskers). ^x^*P* < 0.05 for the vv-ECMO vs. control group values (Mann–Whitney test).

During the post-ECMO period, the RAF (Figure 3A) significantly decreased in the vv-ECMO group relative to the control group. Parallel to the drop in the renal arterial flow, the average hour diuresis fell during the observation period (Figure 3B).

### Changes in Total Hemoglobin Concentration and Hematocrit

The tHb and Hct values decreased early, by the 6th of vv-ECMO treatment and remained significantly lower as compared to the control group until the end of the observation period (Table 1).

**TABLE 1:** The effects of vv-ECMO on blood total hemoglobin concentration (tHb) [g/dL] and hematocrit (Hct) [%]. The table demonstrates the median values and the 25th and 75th percentiles.

tHb			
Time (h)	Parameters	Control	vv-ECMO
0	Median p25; p75	11.3 9.8; 11.8	10.7 8.9; 11.5
6	Median p25; p75	9.3 8.6; 10.9	6.4 x 5.5; 6.8
12	Median p25; p75	9.3 8.3; 10	7.1 x 6.2; 8.1
18	Median p25; p75	9.2 8.1; 9.3	6.4 *x 5.5; 7.2
24	Median p25; p75	8.9 8.7; 1.40	5.5 *x 4.2; 6.8
30	Median p25; p75	9.6 9.2; 10.4	5.5 *x 4.2; 6.7
**Hct**			
0	Median p25; p75	32.7 28.4; 34	30.9 30.1; 34.1
6	Median p25; p75	26 25.4; 33.1	17.8 x 15.2; 18.3
12	Median p25; p75	24.5 23.2; 30.7	17.6 x 16.9; 21.1
18	Median p25; p75	22 20.8; 23	15.1 *x 13.9; 17.9
24	Median p25; p75	23 22; 27,3	14.7 *x 9.9; 19.3
30	Median p25; p75	25.7 23; 27	14.7 *x 11.5; 17.9

*p < 0.05 vs. baseline values; x p < 0.05 vs. control group.

### Histological Changes in the Kidneys

**FIGURE 4:**
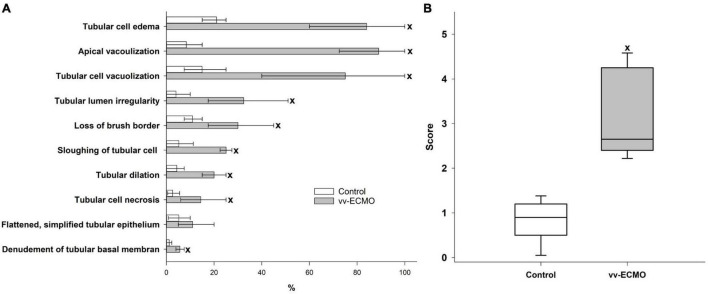
The extent of the renal histological changes **(A)** in the control group (empty column) and vv-ECMO group (gray column). The plots demonstrate the median (top line of the column) and the 25th and 75th percentiles (whiskers). ^x^*P* < 0.05 for the vv-ECMO vs. control group values (Mann–Whitney test). Changes in the renal histology score **(B)** in the control group (empty box) and vv-ECMO group (gray box). The plots demonstrate the median (horizontal line in the box), the 25th and 75th percentiles, and the range of data (whiskers). ^x^*P* < 0.05 for the vv-ECMO vs. control group values (Mann–Whitney test).

Changes in renal hemodynamics and function were accompanied by histological changes in the kidneys. The occurrence ratio of all the histological features under examination was significantly higher in the vv-ECMO group (Figure 4A). A kidney-specific injury score was used to summarize and quantify the qualitative histological alterations, which demonstrated significantly increased injury in the vv-ECMO group (Figure 4B), pointing to the net damage of the renal tissue.

### Renal Tissue Damage: Biochemical Markers

**FIGURE 5:**
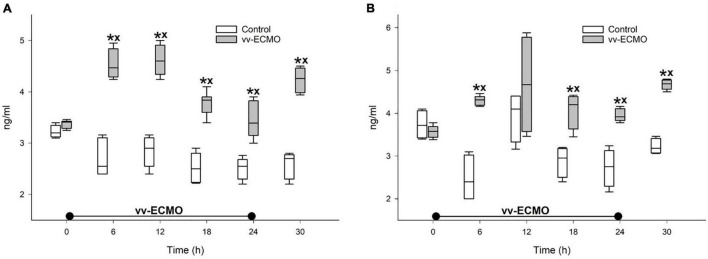
Changes in the neutrophil gelatinase-associated lipocalin levels in the urine **(A)** and plasma **(B)** in the control group (empty box) and vv-ECMO group (gray box). The plots demonstrate the median (horizontal line in the box), the 25th and 75th percentiles, and the range of data (whiskers). *P < 0.05 for groups vs. baseline values (Friedman and Dunn tests), ^x^*P* < 0.05 for the vv-ECMO vs. control group values (Mann–Whitney test).

Renal tissue damage was also characterized by a rise in NGAL levels in both the urine (Figure 5A) and plasma samples (Figure 5B). Compared to the control group, this kidney injury marker increased from the sixth hour of vv-ECMO and remained elevated during the post-ECMO phase until the end of the observation period.

**FIGURE 6:**
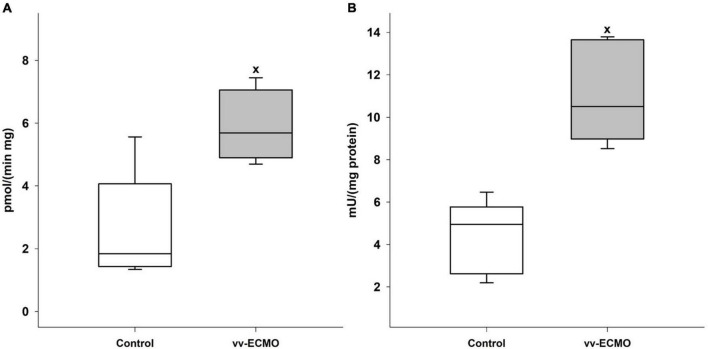
Changes in the xanthine-oxidoreductase **(A)** and myeloperoxidase **(B)** enzyme activities in the renal tissue in the control group (empty box) and vv-ECMO group (gray box). The plots demonstrate the median (horizontal line in the box), the 25th and 75th percentiles, and the range of data (whiskers). ^x^*P* < 0.05 for the vv-ECMO vs. control group values (Mann–Whitney test).

We also demonstrated a significant growth in enzyme activity in two enzymes, which may greatly contribute to oxidative stress in the kidneys. Notably, relative to the control group, both XOR (Figure 6A) and MPO (Figure 6B) activity was elevated in kidney tissue samples that were taken at the end of the experiments.

### Changes in Mitochondrial Oxygen Consumption

**FIGURE 7:**
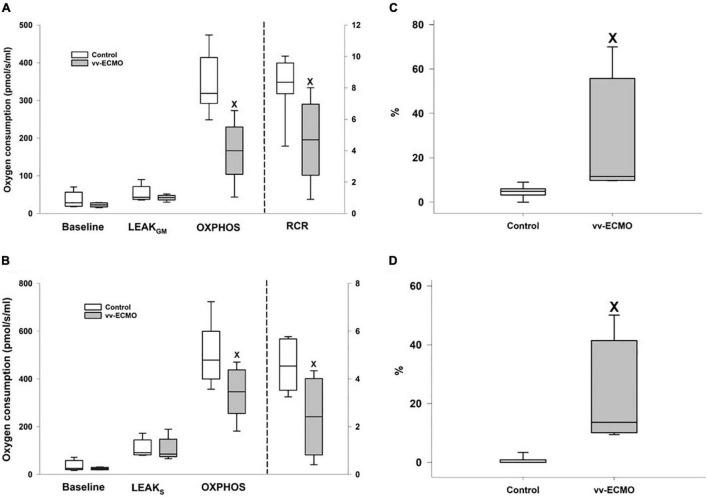
Changes in baseline and LEAK_GM_ respirations, complex I-linked oxidative phosphorylation capacities (OXPHOS; left axis) and respiratory acceptor control ratios (RCR; right axis) in the control (empty box) and vv-ECMO (gray box) groups **(A)**. Changes in baseline and LEAK_S_ respirations, complex II-linked oxidative phosphorylation capacities (OXPHOS; left axis) and respiratory acceptor control ratios (RCR; right axis) in the control (empty box) and vv-ECMO (gray box) groups **(B)**. Changes in the complex I-linked **(C)** and complex II-linked **(D)** oxygen flux following addition of cytochrome c in the control (empty box) and vv-ECMO (gray box) groups. The plots demonstrate the median (horizontal line in the box), the 25th and 75th percentiles, and the range of data (whiskers). ^x^*P* < 0.05 for the vv-ECMO vs. control group values (Mann–Whitney test).

In comparison with the controls, vv-ECMO tissue samples showed a significant drop in complex I- and complex II-linked OXPHOS capacities and in RCR values (Figures 7A,B). In addition, outer membrane permeability rose in these animals, indicated by significantly higher O_2_ flux following addition of cytochrome c (Cytc%; Figures 7C,D). However, baseline respiration or respiration in the presence of complex I- and complex II-linked substrates (LEAK_GM_ and LEAKs; in a non-phosphorylating resting state) did not change significantly (Figures 7A,B).

## Discussion

The clinical use of vv-ECMO is a subject of ongoing debate. Although the improved mortality of patients with severe ARDS and vv-ECMO treatment has been demonstrated (during both the H1N1 and COVID-19 pandemics) (13), the technique still suffers from shortcomings and is accompanied by serious complications, including AKI (13). Nevertheless, it is difficult to outline the direct role of vv-ECMO treatment in clinical setups, as patients already have serious underlying diseases and investigations in healthy humans are impossible. An important aim of preclinical research is to recapitulate the human situation with model experiments. In this study, we have therefore focused on the establishment of a large animal model that makes the examination of the pathomechanism of vv-ECMO-induced AKI feasible. We chose pigs due to the translational similarities to humans, including comparable respiratory physiology and activity of pro-inflammatory enzymes. We also selected AKI as a primary subject, as it can be an important component of multiorgan failure and one of the most important risk factors of mortality among vv-ECMO patients (6, 14).

We were unable to show any differences in MAP and CO between the vv-ECMO-treated and non-treated groups in our model. Nonetheless, HR started to increase in the later phase, including the whole post-ECMO period, in the vv-ECMO group and this was, as expected, accompanied by a decrease in the SV from the 24th hour of the observation period. These changes may point to the initiation of hemodynamic compensatory mechanisms to maintain adequate CO, but this process may also be related to the systemic inflammatory activation induced by the ECMO system. Data from previous *in vitro* studies suggest that IL-1β might be a key component of the process (15). IL-1β affects myocardial contractility in multiple ways, such as modulation of inducible nitric oxide synthase (iNOS) activity (16) and alteration of L-type Ca^2+^ current (17).

The lower average hour diuresis in the vv-ECMO group points to an impaired renal function, which may be the result of a reduced filtration rate caused by the decreased renal perfusion. Indeed, RAF was reduced during the post-ECMO period after vv-ECMO treatment, although there was no difference in CO and MAP values between the two experimental groups. MAP was also kept over 60 mmHg, which is the lowest MAP value necessary to maintain renal function (18). We were not able to obtain RAF data during the ECMO phase for technical reasons. The anticoagulation during ECMO and the need for surgical intervention for the placement of perivascular flow probes around the renal artery would have raised the risk of bleeding. We therefore cut the invasive interventions to a minimum before and during ECMO. Nonetheless, we collected plasma and urine samples during the ECMO phase to measure the levels of kidney injury marker NGAL (19, 20), which provided us with an insight into the condition of the kidneys. These data demonstrate an early increase in both urine and plasma NGAL at 6 h after ECMO initiation. This suggests that the development of AKI starts at a very early stage and precedes systemic hemodynamic changes. Therefore, humoral, inflammatory factors may play a far more important role in the emergence of vv-ECMO-induced AKI than hemodynamic factors.

It should be added that we have already provided evidence for the fall in RAF in another, short-term cardiopulmonary bypass model (21). We explained the phenomenon with the role of superoxide anion generation, as it can raise Ca^2+^ influx into the smooth muscle cells of the renal afferent arterioles, causing vasoconstriction and subsequently reduced renal flow (21, 22). This hypothesis is supported by the heightened activity of MPO and XOR enzymes, which may which greatly contributes to the development of oxidative stress in the renal tissue.

The deterioration of renal tissue perfusion resulted in tubular damage. The histological changes in the kidney samples in the vv-ECMO group correspond to those observed in ischemic kidneys (23). The reduced tHb and Hct could aggravate the effect of the decreased RAF as these factors are key components of the local, tissue oxygen delivery (DO_2_). However, the development of tubular damage is multifactorial, and other causes besides an impaired renal DO_2_, including inflammatory activation and mitochondrial damage, are significant inducers of AKI of other origins (24, 25). Indeed, our study is the first to demonstrate vv-ECMO-linked mitochondrial functional impairment in non-cardiac tissue. Structural and functional changes in cardiac mitochondria have previously been confirmed in pigs following vv-ECMO (26), and now we have found that both complex I- and complex II-linked OXPHOS capacities (an indicator of ATP synthesis) and RCR (respiration coupled to OXPHOS) values were markedly lower in the kidney. Moreover, the integrity of the outer membrane was impaired, indicated by elevated respiration following addition of cytochrome c. The ECMO-related mitochondrial dysfunction may also be linked to the release of pro-inflammatory cytokines, such as TNF-α, IL-1β and IL-6 (27). Ligation of TNF receptors with TNF-α is associated with (1) increased mitochondrial ROS formation at complex I and complex III, (2) inhibition of OXPHOS and ATP depletion *via* tyrosine phosphorylation at subunit I of cytochrome c oxidase, (3) decreased mitochondrial membrane potential and (4) altered mitochondrial integrity (28, 29). Dysfunctional mitochondria generate a high level of reactive oxygen species (ROS), release cell-free mitochondrial DNA (cf-mtDNA) and multiply damage-associated molecular patterns (DAMPs), such as cardiolipin, ATP and cytochrome c (30); all these events may ultimately lead to further mitochondrial and kidney damage. There is also evidence that ECMO affects metabolic flexibility and upregulates pyruvate dehydrogenase kinase-4 (PDK4), a crucial enzyme responsible for the suppression of pyruvate dehydrogenase complex (PDC) (31, 32). PDK4 plays a decisive role in the metabolic shift from OXPHOS to glycolysis; PDK4 downregulation lowered lactate level and boosted ATP generation, whereas PDK4 over-expression increased lactate and reduced ATP level *in vitro* (33). A similar PDK4-related mechanism on mitochondrial respiration cannot be ruled out and may be the subject of further study.

There are a few limitations to discuss. The 24-hour time frame of the vv-ECMO treatment can be considered relatively short from a clinical point of view. We chose this period as it provides a clinically relevant balance between the human situation and the material resources and technical background of *in vivo* large animal studies. The oliguric phase after the start of ECMO occurs mostly in the first 24–48 h, and recovery may start after 48 h, with the diuretic phase following (34). Therefore, we considered the 24-hour treatment suitable to examining the processes that play a role in the early development of vv-ECMO-related AKI. Another important limitation is the lack of data on RAF during vv-ECMO, which could be overcome with the application of non-invasive techniques, such as ultrasound Doppler. Nonetheless, these techniques are far less reliable than perivascular flow probes (35). Also, this might require the repositioning of the animals, which heightens the risk of accidental decannulation or of movement of the cannulas from the ideal position. Finally, it is important to note that the transpulmonary thermodilution technique may be inaccurate during ECMO due to the potential loss of the indicator. Nonetheless, recent studies have demonstrated that the method can be applied during vv-ECMO, especially when the indicator is injected *via* the jugular vein (36, 37).

## Conclusion

We have presented a porcine model suitable for the examination of renal complications of vv-ECMO. The findings show that vv-ECMO for 24 h may induce AKI, which is more likely associated with vv-ECMO-related inflammatory signals than changes in systemic hemodynamics. Oxidative stress and mitochondrial injury are key contributors to the progression that results in structural and functional damage to the kidneys in this scenario.

## Data Availability Statement

The raw data supporting the conclusions of this article will be made available by the authors, without undue reservation.

## Ethics Statement

The animal study was reviewed and approved by National Scientific Ethical Committee on Animal Experimentation (National Competent Authority of Hungary).

## Author Contributions

GB, GV, and DÉ designed the study. AS-B, GV, ZV, GB, GyV, ÁG, NV, ÁH, AR, MG, and DÉ performed the experiments. ZV, GyV, ÁG, NV, ÁH, and MG managed databases. AS-B, GV, and DÉ evaluated data and wrote the manuscript. GV, AN, and AR prepared figures. LJ and AN measured mitochondrial respiratory function and wrote the manuscript. ST-N performed histological evaluation. AS-B, GV, DÉ, and MB supervised and edited the manuscript. All authors contributed to the article and approved the submitted version.

## Conflict of Interest

The authors declare that the research was conducted in the absence of any commercial or financial relationships that could be construed as a potential conflict of interest.

## Publisher’s Note

All claims expressed in this article are solely those of the authors and do not necessarily represent those of their affiliated organizations, or those of the publisher, the editors and the reviewers. Any product that may be evaluated in this article, or claim that may be made by its manufacturer, is not guaranteed or endorsed by the publisher.

## References

[1] Ostermann M Lumlertgul N. Acute kidney injury in ECMO patients. In: Vincent J-L editor. *Annual Update in Intensive Care and Emergency Medicine 2021.* Cham: Springer International Publishing (2021). p. 207–22. 10.1007/978-3-030-73231-8_18

[2] Zangrillo A Landoni G Biondi-Zoccai G Greco M Greco T Frati G A meta-analysis of complications and mortality of extracorporeal membrane oxygenation. *Crit Care Resusc.* (2013) 15:172–8. 23944202

[3] Askenazi DJ Selewski DT Paden ML Cooper DS Bridges BC Zappitelli M Renal replacement therapy in critically ill patients receiving extracorporeal membrane oxygenation. *Clin J Am Soc Nephrol.* (2012) 7:1328–36. 10.2215/CJN.12731211 22498496 PMC5486859

[4] Delmas C Zapetskaia T Conil JM Georges B Vardon-Bounes F Seguin T 3-month prognostic impact of severe acute renal failure under veno-venous ECMO support: importance of time of onset. *J Crit Care.* (2018) 44:63–71. 10.1016/j.jcrc.2017.10.022 29073534

[5] Ostermann M Connor MJ Kashani K. Continuous renal replacement therapy during extracorporeal membrane oxygenation: why, when and how? *Curr Opin Crit Care.* (2018) 24:493–503. 10.1097/MCC.0000000000000559 30325343

[6] Thongprayoon C Cheungpasitporn W Lertjitbanjong P Aeddula NR Bathini T Watthanasuntorn K Incidence and impact of acute kidney injury in patients receiving extracorporeal membrane oxygenation: a meta-analysis. *J Clin Med.* (2019) 8:981. 10.3390/jcm8070981 31284451 PMC6678289

[7] Lin C-Y Chen Y-C Tsai F-C Tian Y-C Jenq C-C Fang J-T RIFLE classification is predictive of short-term prognosis in critically ill patients with acute renal failure supported by extracorporeal membrane oxygenation. *Nephrol Dial Transplant.* (2006) 21:2867–73. 10.1093/ndt/gfl326 16799171

[8] De Corte W Dhondt A Vanholder R De Waele J Decruyenaere J Sergoyne V Long-term outcome in ICU patients with acute kidney injury treated with renal replacement therapy: a prospective cohort study. *Crit Care.* (2016) 20:256. 10.1186/s13054-016-1409-z 27520553 PMC4983760

[9] Husain-Syed F Ricci Z Brodie D Vincent J-L Ranieri VM Slutsky AS Extracorporeal organ support (ECOS) in critical illness and acute kidney injury: from native to artificial organ crosstalk. *Intensive Care Med.* (2018) 44:1447–59. 10.1007/s00134-018-5329-z 30043276

[10] Beckman JS Parks DA Pearson JD Marshall PA Freeman BA. A sensitive fluorometric assay for measuring xanthine dehydrogenase and oxidase in tissues. *Free Radic Biol Med.* (1989) 6:607–15. 10.1016/0891-5849(89)90068-3 2753392

[11] Kuebler WM Abels C Schuerer L Goetz AE. Measurement of neutrophil content in brain and lung tissue by a modified myeloperoxidase assay. *Int J Microcirc Clin Exp Spons Eur Soc Microcirc.* (1996) 16:89–97. 10.1159/000179155 8737712

[12] Dupont WD Plummer WD. Power and sample size calculations: a review and computer program. *Control Clin Trials.* (1990) 11:116–28. 10.1016/0197-2456(90)90005-m 2161310

[13] Kim JH Pieri M Landoni G Scandroglio AM Calabrò MG Fominskiy E Venovenous ECMO treatment, outcomes, and complications in adults according to large case series: a systematic review. *Int J Artif Organs.* (2021) 44:481–8. 10.1177/0391398820975408 33259258

[14] Mou Z He J Guan T Chen L. Acute kidney injury during extracorporeal membrane oxygenation: VA ECMO versus VV ECMO. *J Intensive Care Med.* (2021). [Online ahead of print]. 10.1177/08850666211035323 34397300

[15] Adrian K Mellgren K Skogby M Friberg LG Mellgren G Wadenvik H. Cytokine release during long-term extracorporeal circulation in an experimental model. *Artif Organs.* (1998) 22:859–63. 10.1046/j.1525-1594.1998.06121.x 9790084

[16] Stein B Frank P Schmitz W Scholz H Thoenes M. Endotoxin and cytokines induce direct cardiodepressive effects in mammalian cardiomyocytes via induction of nitric oxide synthase. *J Mol Cell Cardiol.* (1996) 28:1631–9. 10.1006/jmcc.1996.0153 8877773

[17] El Khoury N Mathieu S Fiset C. Interleukin-1β reduces L-type Ca^2+^ current through protein kinase C∈ activation in mouse heart. *J Biol Chem.* (2014) 289:21896–908. 10.1074/jbc.m114.549642 24936064 PMC4139208

[18] Redfors B Bragadottir G Sellgren J Swärd K Ricksten SE. Effects of norepinephrine on renal perfusion, filtration and oxygenation in vasodilatory shock and acute kidney injury. *Intensive Care Med.* (2011) 37:60–7. 10.1007/s00134-010-2057-4 20949349

[19] Bulluck H Maiti R Chakraborty B Candilio L Clayton T Evans R Neutrophil gelatinase-associated lipocalin prior to cardiac surgery predicts acute kidney injury and mortality. *Heart.* (2018) 104:313–7. 10.1136/heartjnl-2017-311760 28794136 PMC5861395

[20] Lima C de Paiva Haddad LB de Melo PDV Malbouisson LM do Carmo LPF D’Albuquerque LAC Early detection of acute kidney injury in the perioperative period of liver transplant with neutrophil gelatinase-associated lipocalin. *BMC Nephrol.* (2019) 20:367. 10.1186/s12882-019-1566-9 31615452 PMC6794911

[21] Bari G Érces D Varga G Szűcs S Varga Z Bogáts G Methane inhalation reduces the systemic inflammatory response in a large animal model of extracorporeal circulation. *Eur J Cardiothorac Surg.* (2019) 56:135–42. 10.1093/ejcts/ezy453 30649294

[22] Vogel PA Yang X Moss NG Arendshorst WJ. Superoxide enhances Ca^2+^ entry through L-type channels in the renal afferent arteriole. *Hypertension.* (2015) 66:374–81. 10.1161/HYPERTENSIONAHA.115.05274 26034201 PMC4499026

[23] van Smaalen TC Ellis SR Mascini NE Siegel TP Cillero-Pastor B Hillen LM Rapid identification of ischemic injury in renal tissue by mass-spectrometry imaging. *Anal Chem.* (2019) 91:3575–81. 10.1021/acs.analchem.8b05521 30702282 PMC6581420

[24] Bhatia D Capili A Choi ME. Mitochondrial dysfunction in kidney injury, inflammation, and disease: potential therapeutic approaches. *Kidney Res Clin Pract.* (2020) 39:244–58. 10.23876/j.krcp.20.082 32868492 PMC7530368

[25] Ishimoto Y Inagi R. Mitochondria: a therapeutic target in acute kidney injury. *Nephrol Dial Transplant.* (2016) 31:1062–9. 10.1093/ndt/gfv317 26333547

[26] Shen J Yu W Shi J Chen Q Hu Y Zhang J Effect of venovenous extracorporeal membrane oxygenation on the heart in a healthy piglet model. *J Cardiothorac Surg.* (2013) 8:163. 10.1186/1749-8090-8-163 23805777 PMC3706349

[27] Shen J Yu W Chen Q Shi J Hu Y Zhang J Continuous renal replacement therapy (CRRT) attenuates myocardial inflammation and mitochondrial injury induced by venovenous extracorporeal membrane oxygenation (VV ECMO) in a healthy piglet model. *Inflammation.* (2013) 36:1186–93. 10.1007/s10753-013-9654-7 23700116

[28] Kastl L Sauer SW Ruppert T Beissbarth T Becker MS Süss D TNF-α mediates mitochondrial uncoupling and enhances ROS-dependent cell migration via NF-κB activation in liver cells. *FEBS Lett.* (2014) 588:175–83. 10.1016/j.febslet.2013.11.033 24316229

[29] Samavati L Lee I Mathes I Lottspeich F Hüttemann M. Tumor necrosis factor α inhibits oxidative phosphorylation through tyrosine phosphorylation at subunit I of cytochrome c oxidase. *J Biol Chem.* (2008) 283:21134–44. 10.1074/jbc.M801954200 18534980 PMC3258931

[30] Thurairajah K Briggs GD Balogh ZJ. The source of cell-free mitochondrial DNA in trauma and potential therapeutic strategies. *Eur J Trauma Emerg Surg.* (2018) 44:325–34. 10.1007/s00068-018-0954-3 29633007 PMC6002458

[31] Kajimoto M O’Kelly Priddy CM Ledee DR Xu C Isern N Olson AK Extracorporeal membrane oxygenation promotes long chain fatty acid oxidation in the immature swine heart in vivo. *J Mol Cell Cardiol.* (2013) 62:144–52. 10.1016/j.yjmcc.2013.05.014 23727393 PMC3739709

[32] Zhang S Hulver MW McMillan RP Cline MA Gilbert ER. The pivotal role of pyruvate dehydrogenase kinases in metabolic flexibility. *Nutr Metab.* (2014) 11:10. 10.1186/1743-7075-11-10 24520982 PMC3925357

[33] Liu X Zuo R Bao Y Qu X Sun K Ying H. Down-regulation of PDK4 is critical for the switch of carbohydrate catabolism during syncytialization of human placental trophoblasts. *Sci Rep.* (2017) 7:8474. 10.1038/s41598-017-09163-8 28814762 PMC5559526

[34] Vyas A Bishop MA. *Extracorporeal Membrane Oxygenation in Adults.* Treasure Island, FL: StatPearls Publishing (2022). 35015451

[35] Wan L Yang N Hiew C-Y Schelleman A Johnson L May C An assessment of the accuracy of renal blood flow estimation by Doppler ultrasound. *Intensive Care Med.* (2008) 34:1503–10. 10.1007/s00134-008-1106-8 18408915

[36] Herner A Lahmer T Mayr U Rasch S Schneider J Schmid RM Transpulmonary thermodilution before and during veno-venous extra-corporeal membrane oxygenation ECMO: an observational study on a potential loss of indicator into the extra-corporeal circuit. *J Clin Monit Comput.* (2020) 34:923–36. 10.1007/s10877-019-00398-6 31691149

[37] Lahmer T Mayr U Rasch S Batres Baires G Schmid RM Huber W. In-parallel connected intermittent hemodialysis through ECMO does not affect hemodynamic parameters derived from transpulmonary thermodilution. *Perfusion.* (2017) 32:702–5. 10.1177/0267659117707816 28440110

